# Synthetic Hydrogels Incorporating Hydrolytic/Nonhydrolytic Macromer Ratios Exhibit Improved Tunability of In Vivo Degradation and Immune Responses

**DOI:** 10.1002/adhm.202502475

**Published:** 2025-10-01

**Authors:** Michael D. Hunckler, Sophia Kioulaphides, Karen E. Martin, Angelica L. Torres, Graham F. Barber, Stephen W. Linderman, Rebecca S. Schneider, Andrés J. García

**Affiliations:** ^1^ Woodruff School of Mechanical Engineering Georgia Institute of Technology 801 Ferst Dr NW Atlanta GA 30332 USA; ^2^ Petit Institute for Bioengineering and Bioscience Georgia Institute of Technology 315 Ferst Dr NW Atlanta GA 30332 USA; ^3^ Wallace H. Coulter Department of Biomedical Engineering Georgia Institute of Technology 313 Ferst Dr NW Atlanta GA 30332 USA; ^4^ Emory University School of Medicine Emory University 100 Woodruff Cir Atlanta GA 30322 USA; ^5^ School of Chemical and Biomolecular Engineering Georgia Institute of Technology 311 Ferst Dr NW Atlanta GA 30332 USA

**Keywords:** hydrogels, hydrolytic degradability, poly(ethylene glycol), tunability

## Abstract

Proteolytically degradable hydrogels are widely used as delivery carriers in regenerative medicine. However, the in vivo degradation rate of these materials is difficult to control because of site‐specific enzymatic activity, implant design, and disease state, impairing tissue regeneration. Hydrogels with crosslinks that degrade hydrolytically offer an alternate route to tune in vivo degradation profile. In this study, a synthetic 4‐arm maleimide‐terminated poly(ethylene glycol) (PEG‐4MAL) hydrogel system that combines hydrolytic ester‐linked PEG‐4MAL (PEG‐4eMAL) macromer with nondegradable amide‐linked PEG‐4MAL (PEG‐4aMAL) macromer in various stoichiometric ratios to tune the degradability rate is engineered. The macromers are crosslinked with dithiothreitol (DTT) via thiol‐maleimide click reaction. Rheological analysis shows that a family of PEG‐4eMAL/PEG‐4aMAL hydrogels has equivalent mechanical properties, but increasing the PEG‐4eMAL content increases the rate of degradation in vitro and in vivo. PEG‐4eMAL/PEG‐4aMAL hydrogels support high viability of encapsulated human cells. Notably, the ratio of PEG‐4eMAL/PEG‐4aMAL modulates local immune cell recruitment when implanted in the subcutaneous space. These results establish the use of PEG‐4eMAL/PEG‐4aMAL hydrogels as a hydrolytically degradable platform to tune in vivo degradation and immune responses.

## Graphical abstract

A synthetic 4‐arm maleimide‐terminated poly(ethylene glycol) (PEG‐4MAL) hydrogel system that combines hydrolytic ester‐linked macromer (PEG‐4eMAL) with nondegradable amide‐linked macromer (PEG‐4aMAL) in various stoichiometric ratios to tune the degradability rate. The macromers are crosslinked with dithiothreitol via thiol‐maleimide click reaction. The family of PEG‐4eMAL/PEG‐4aMAL hydrogels has equivalent mechanical properties, but increasing the PEG‐4eMAL content increases the rate of degradation in vitro and in vivo and alters local immune cell recruitment.

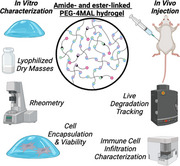

## 1 Introduction

Degradable hydrogels are used extensively for tissue regeneration, cell engraftment, drug delivery, and many other biomedical applications.^[^ ^1^, ^2^ ^]^ Protease‐degradable hydrogels have been developed to promote in vivo cell infiltration and tissue repair. These hydrogels are typically crosslinked with peptides that are susceptible to degradation by specific proteases such as matrix metalloproteinases (MMPs), and varying the number of these degradable crosslinks can be used to tune degradation rate.^[^ ^3^ ^]^ This platform has been explored for its ability to promote cell engraftment and vascularization in multiple animal models.^[^ ^1^, ^4^, ^5^, ^6^ ^]^ However, proteolytically degradable hydrogels do not exhibit predictable degradation rates in vivo^[^ ^7^ ^]^ because of site‐specific differences in enzymatic activity, implant design (e.g., volume, configuration), and disease state (e.g., degree of inflammation).

The implantation of a biomaterial induces an injury at the site of transplantation, initiating an inflammatory response that ultimately determines the biomaterial's performance.^[^ ^8^, ^9^ ^]^ The site of implantation has a major effect on this outcome. For example, areas of the body under constant mechanical stress or high protease activity result in faster degradation of the biomaterial and subpar tissue regeneration.^[^ ^10^ ^]^ Furthermore, the disease state of the host greatly influences the local immune environment and therefore the performance of the implant. For instance, a diabetic patient will have impaired wound healing, whereas patients with osteoporosis have an increased risk of implant loosening due to reduced osseointegration.^[^ ^11^ ^]^ In addition, the size and shape of a hydrogel can modulate its degradation. Implants with larger surface area/volume ratios degrade faster than implants with smaller ratios due to higher access to proteases.^[^ ^12^ ^]^ Finally, encapsulating cells within these biomaterials can modulate the degradability of hydrogels as many cell types secrete proteases.^[^ ^13^, ^14^ ^]^

Common strategies to tune the degradation rate of proteolytically degradable hydrogels involve altering 1) polymer density and/or 2) ratio of degradable/nondegradable crosslinking peptides.^[^ ^7^, ^15^, ^16^, ^17^ ^]^ Both approaches have limitations. When modulating polymer density, hydrogel mechanical properties will also vary considerably, and thus impact cell behaviors and host‐biomaterial interactions. Changing the ratio of proteolytically degradable/nondegradable crosslinks can lead to implants with varying degradation rates dependent on the local concentration of different proteases. To overcome these limitations of proteolytically degradable hydrogels, several alternative methods of biomaterial degradation have been explored, including hydrolysis, oxidation, and physical deterioration via water swelling and mechanical wearing.^[^ ^18^ ^]^ In hydrolytic degradation, polymer chains (e.g., polyesters, polyorthesters) react with water leading to chain scission.^[^ ^18^ ^]^ The use of hydrolytically degradable polymers as crosslinkers or elements of the backbone in the hydrogel network has been studied in natural^[^ ^19^, ^20^ ^]^ and synthetic biomaterials.^[^ ^21^, ^22^, ^23^, ^24^ ^]^ The most common forms of hydrolytic degradation reported in the literature are oxidation of natural polymers, incorporation of dithiol crosslinkers in synthetic hydrogel platforms, use of peptides such as N,N‐bisacryloxyethyl amide as crosslinkers to allow for enzymatic hydrolysis, and incorporation of ester linkages into synthetic hydrogel backbones.^[^ ^19^, ^23^, ^25^, ^26^, ^27^, ^28^, ^29^, ^30^, ^31^, ^32^, ^33^, ^34^, ^35^ ^]^ This body of literature has demonstrated the ability to alter the composition of hydrogels to increase degradation rates, regardless of the application of interest. In this study, we engineered a synthetic 4‐arm maleimide‐terminated poly(ethylene glycol) (PEG‐4MAL) hydrogel system that combines hydrolytically degradable, ester‐linked PEG‐4MAL (PEG‐4eMAL) macromer with nondegradable amide‐linked PEG‐4MAL (PEG‐4aMAL) macromer in a range of stoichiometric ratios to tune degradability. Increasing the PEG‐4eMAL content in PEG‐4eMAL/PEG‐4aMAL hydrogels increased the in vitro and in vivo degradation rate of hydrogels while maintaining equivalent mechanical properties. Notably, the ratio of PEG‐4eMAL/PEG‐4aMAL modulated local immune cell recruitment when implanted in the subcutaneous space. These results establish the use of PEG‐4eMAL/PEG‐4aMAL hydrogels as a hydrolytically degradable platform to tune in vivo degradation and immune responses.

## 2 Results

### 2.1 PEG‐4eMAL/PEG‐4aMAL Hydrogels Exhibit Equivalent Mechanical Properties

**Figure 1:**
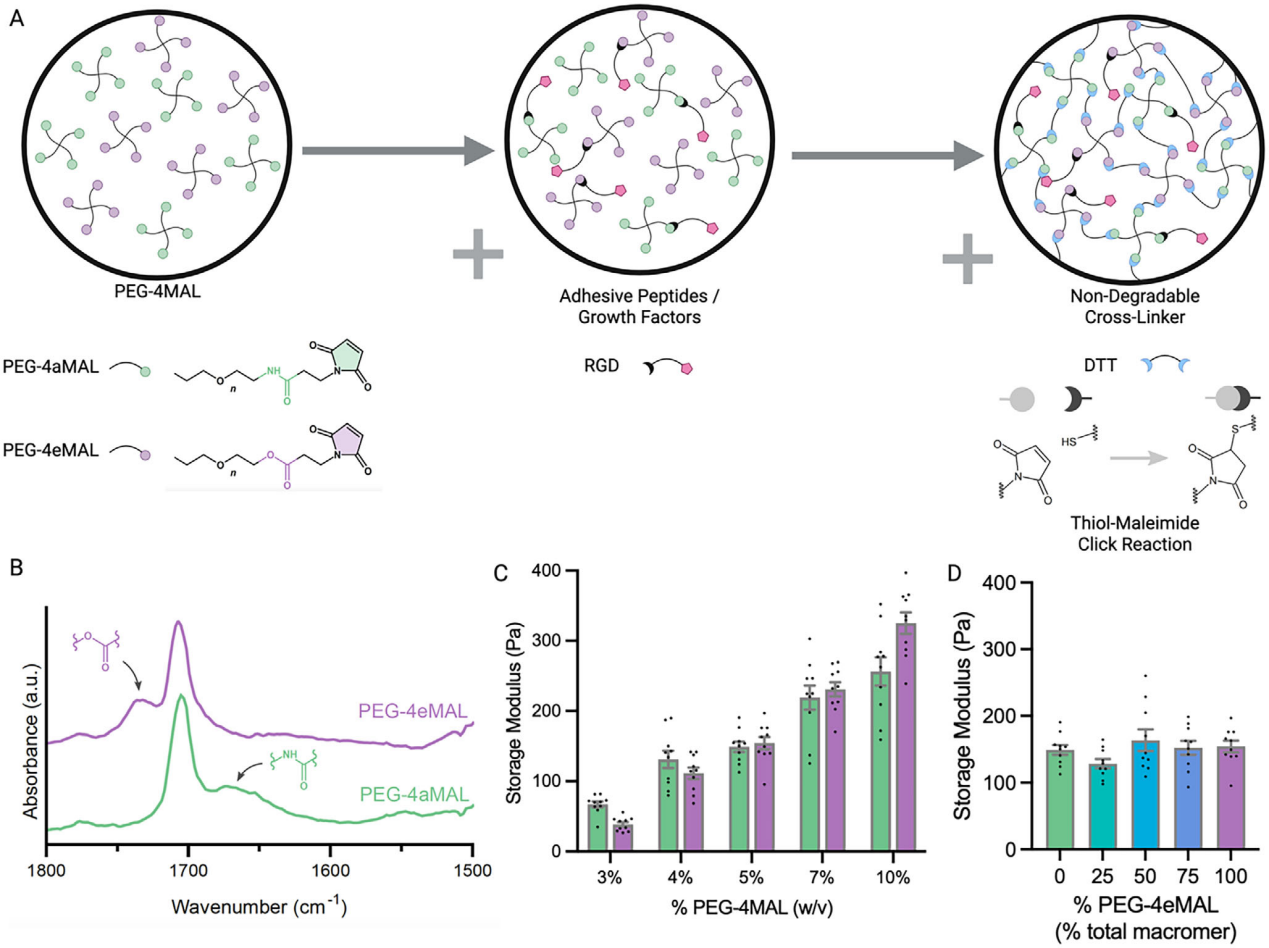
Synthesis and characterization of PEG hydrogels with varying ratios of ester/amide linkages. A) 4‐arm PEG macromer, terminated with amide‐maleimide (green) or ester‐maleimide (purple), are mixed and reacted with cysteine‐containing adhesive peptides and crosslinked with DTT. The thiol‐maleimide click reaction is the basis for network formation. B) FTIR spectra of lyophilized hydrogels (without adhesive peptides) reveal ester and amide peaks. C) Equivalent mechanical properties of 100% PEG‐4eMAL and 100% PEG‐4aMAL gels at each polymer density (*p* = 0.32, two‐way ANOVA). D) Increasing the macromer content of PEG‐4eMAL did not change the mechanical properties in 5% (w/v) PEG‐4MAL hydrogels (*p* = 0.21; one‐way ANOVA). All data is presented as mean ± standard error of the mean (s.e.m.), with *n* = 10 gels/group.

We have previously synthesized hydrogels based on 20 kDa PEG‐4aMAL and crosslinked with protease‐degradable peptides.^[^ ^1^, ^4^, ^5^, ^6^ ^]^ To create an analogous hydrolytically degradable platform, we employed a 20 kDa PEG‐4eMAL macromer (**Figure** **1**A). We hypothesized that for a fixed total polymer density these two macromers with equivalent molecular weight, as well as mixtures with varying ratios of PEG‐4aMAL/PEG‐4eMAL macromer, form mechanically equivalent hydrogels. To promote cell adhesive interactions, adhesive peptides such as the arginine‐glycine‐aspartic acid (RGD) peptide derived from fibronectin were tethered to the macromer. To generate hydrogels, macromer was crosslinked with dithiothreitol (DTT) via thiol‐maleimide click reaction. Fourier Transform infrared spectroscopy (FTIR) analysis confirmed the presence of the amide group in PEG‐4aMAL hydrogels and the ester group in PEG‐4eMAL hydrogels (Figure 1B). For rheological analyses, hydrogels synthesized at 3, 4, 5, 7, and 10 weight/volume% (w/v%) polymer density were measured at a frequency sweep from 1 to 10 Hz. There were no differences in gelation among PEG‐4aMAL/PEG‐4eMAL formulations; all hydrogel formulations fully gelled in <5 min. Storage modulus increased with polymer density, and there were no differences between PEG‐4aMAL and PEG‐4eMAL hydrogels (Figure 1C). For all formulations, loss moduli were <10% of the storage moduli, indicating that these hydrogels behave as elastic solids (Figure S1, Supporting Information). Furthermore, for a fixed polymer density (5 w/v%), hydrogels consisting of defined mixtures of PEG‐4eMAL and PEG‐4aMAL macromer (0%, 25%, 50%, 75%, and 100% PEG‐4eMAL) — which is expected to alter the number of hydrolytically degradable crosslinks — showed no differences in mechanical properties (Figure 1D). These results demonstrate that hydrogels with varying ratios of PEG‐4eMAL/PEG‐4aMAL macromers but equivalent mechanical properties are easily synthesized.

### 2.2 Ester Linkage in PEG‐4MAL Hydrogels Increases In Vitro Degradation Rate

**Figure 2:**
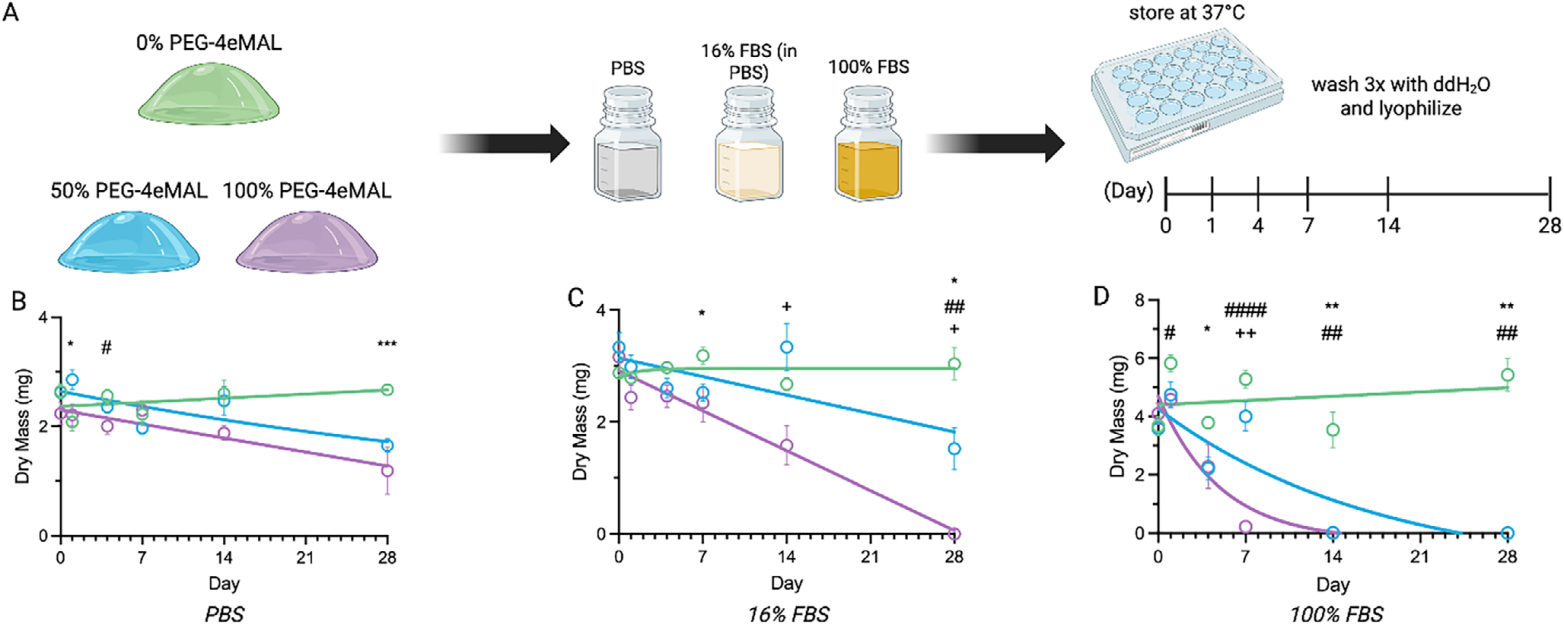
In vitro degradation of PEG‐4MAL hydrogels in serum‐containing buffers. A) Schematic of PEG‐4eMAL/PEG‐4aMAL hydrogel formulations, reagents for incubation, and time points of gel lyophilization (*n* = 5 gels/group in each buffer). Degradation across 28 days in the three media formulations; statistical differences in dry mass are denoted by the following symbols: ^*^(0% vs 50%), ^#^(0% vs 100%), ^+^(50% vs 100%). B) PBS: Day 1 (^*^ *p* = 0.0313), Day 4 (^#^ *p* = 0.0466), Day 28 (^***^ *p* = 0.0008). C) 16% FBS: Day 7 (^*^ *p* = 0.0409), Day 14 (^+^ *p* = 0.0306), Day 28 (^*^ *p* = 0.0330, ^##^ *p* = 0.0011, ^+^ *p* = 0.0337). D) 100% FBS: Day 1 (^#^ *p* = 0.0216), Day 4 (^*^ *p* = 0.0261), Day 7 (^####^ *p* < 0.0001, ^++^ *p* = 0.0022), Day 14 (^**^ *p* = 0.0097, ^##^ *p* = 0.0096), Day 28 (^**^ *p* = 0.0014, ^##^ *p* = 0.0014). Results displayed as mean ± s.e.m. with a nonlinear line of best fit. Data was analyzed with two‐way ANOVA.

We next evaluated the degradation profile for PEG‐4MAL hydrogels in serum‐free and serum‐containing buffers. PEG‐4MAL hydrogels with 0%, 50%, and 100% PEG‐4eMAL macromer were functionalized with RGD and crosslinked with DTT. Crosslinked hydrogels were placed in phosphate‐buffered saline (PBS), 16% fetal bovine serum (FBS) in PBS (mimicking the concentration of FBS in culture media), and 100% FBS to swell. Hydrogels were maintained under cell culture conditions (37 °C, 5% CO_2_), washed 3X with distilled water (dH_2_O) to remove excess proteins and salts that could influence dry mass measurements, and lyophilized at selected time points over 28 days (**Figure** **2**A). Dry mass of the remaining polymer was measured and analyzed to assess polymer degradation. Prior work showed that polymer degradation can be assessed by measuring dried mass or storage modulus (rheometry) with similar results.^[^ ^35^ ^]^ None of the hydrogels fully degraded in 28 days in PBS at 37 °C, although those with 50% and 100% PEG‐4eMAL displayed partial degradation (Figure 2B). In contrast, when incubated in PBS at room temperature, no degradation was observed across 28 days, showing that temperature accelerates degradation (Figure S2A,B, Supporting Information). The 100% PEG‐4eMAL hydrogels fully degraded by day 28 in 16% FBS, exhibiting higher degradation than 0% PEG‐4eMAL hydrogels (Figure 2C). When hydrogels were incubated in 100% FBS, those with 100% PEG‐4eMAL macromer completely degraded by day 7, hydrogels with 50% PEG‐4eMAL macromer fully degraded by day 14, whereas 0% PEG‐4eMAL hydrogels showed no signs of degradation (Figure 2D). We attribute the accelerated degradation of the PEG‐4eMAL macromer in serum to enzyme‐catalyzed hydrolysis from serum esterases.^[^ ^36^ ^]^ To gain further insights into the effects of serum, 100% PEG‐4aMAL and 100% PEG‐4eMAL hydrogels were formulated and incubated in heat‐inactivated murine and fetal bovine serum or naïve (no heat inactivation) murine serum (Figure S2C, Supporting Information). Within 24 h, 100% PEG‐4eMAL hydrogels completely degraded in naïve serum, whereas those in heat‐inactivated sera exhibited no degradation (Figure S2D,E, Supporting Information). This result supports the hypothesis that enzyme‐catalyzed degradation is the primary driver of accelerated degradation, although further analysis is needed to identify the active protease(s). Taken together, these data show that serum accelerates the in vitro degradation of hydrogels containing PEG‐4eMAL, and the PEG‐4eMAL/PEG‐4aMAL ratio controls the in vitro degradation rate of these hydrogels.

### 2.3 PEG‐4eMAL/PEG‐4aMAL Hydrogels Support In Vitro Human Cell Viability

**Figure 3:**
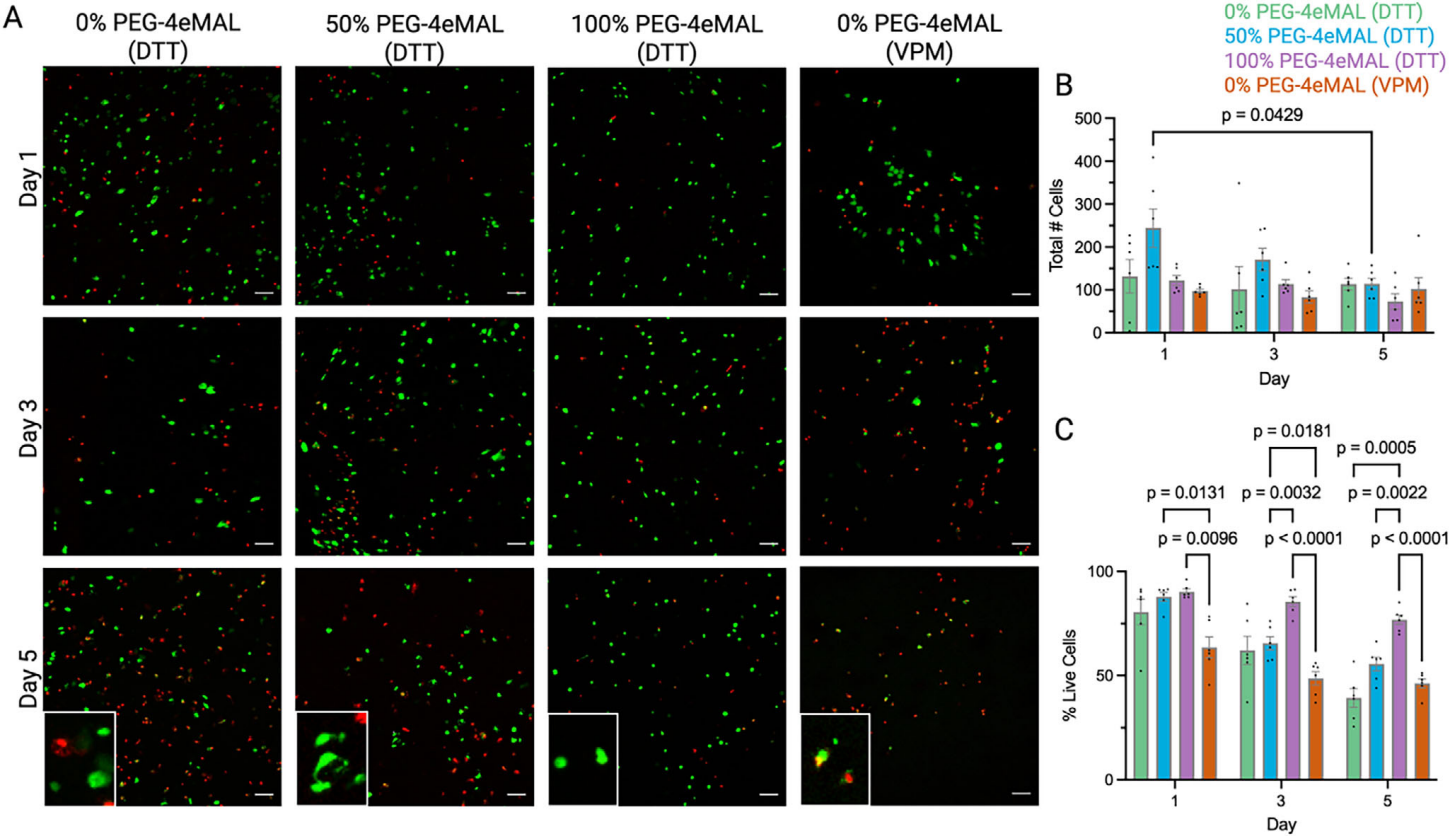
In vitro viability of human mesenchymal stromal cells in PEG‐4MAL hydrogels. A) Images of hMSCs stained with Calcein AM (green) and Ethidium Homodimer‐1 (red) to denote live and dead cells, respectively, across different PEG‐4MAL formulations and time points (*n* = 4/group within time point). Inset shows higher magnification images. Quantification of B) the total number of cells and C) the percentage of live cells across the PEG‐4MAL formulations over time. Data presented as bar plots (mean ± s.e.m.) with individual data points and analyzed with two‐way ANOVA. Scale bar = 100 µm.

We next examined the cytocompatibility of the PEG‐4eMAL/PEG‐4aMAL hydrogel system as this platform is attractive for cell delivery applications. Human mesenchymal stromal cells (hMSCs) have been extensively evaluated in regenerative medicine applications,^[^ ^16^, ^37^, ^38^, ^39^, ^40^, ^41^, ^42^ ^]^ making them a relevant cell type to investigate. Cells were embedded at a density of 1×10^6^ cells mL^−1^ in RGD‐functionalized (to support cell adhesion) 5 w/v% PEG hydrogels synthesized with 0%, 50%, or 100% PEG‐4eMAL macromer and crosslinked with DTT; RGD‐functionalized PEG‐4aMAL hydrogels crosslinked with protease‐degradable peptide (VPM) were used as a reference. Hydrogels were incubated in complete culture media containing 16% FBS and stained for live and dead cells at 1, 3, and 5 days after embedding (**Figure** **3**A). Images across conditions and time points show more cell death by day 5 in the proteolytically degradable and non‐degradable hydrogels compared to those containing PEG‐4eMAL macromer. Quantification of these images corroborated these observations; total numbers of cells across the hydrogel groups at each timepoint were comparable (Figure 3B), and by day 5 the percentage of live cells was significantly higher in 100% PEG‐4eMAL hydrogels compared to all other groups (Figure 3C). We attribute the increased cell viability in the PEG‐4eMAL hydrogels to increased network degradation that allows enhanced cell spreading, corroborating previous reports.^[^ ^16^, ^25^, ^43^, ^44^, ^45^, ^46^ ^]^ However, further studies can be performed to better characterize hMSC fate in the PEG‐4eMAL‐based hydrogel network, including matrix deposition, secretory functions, and differentiation.^[^ ^47^, ^48^, ^49^ ^]^

### 2.4 PEG‐4eMAL/PEG‐4aMAL Ratio Controls In Vivo Degradation Rate

**Figure 4:**
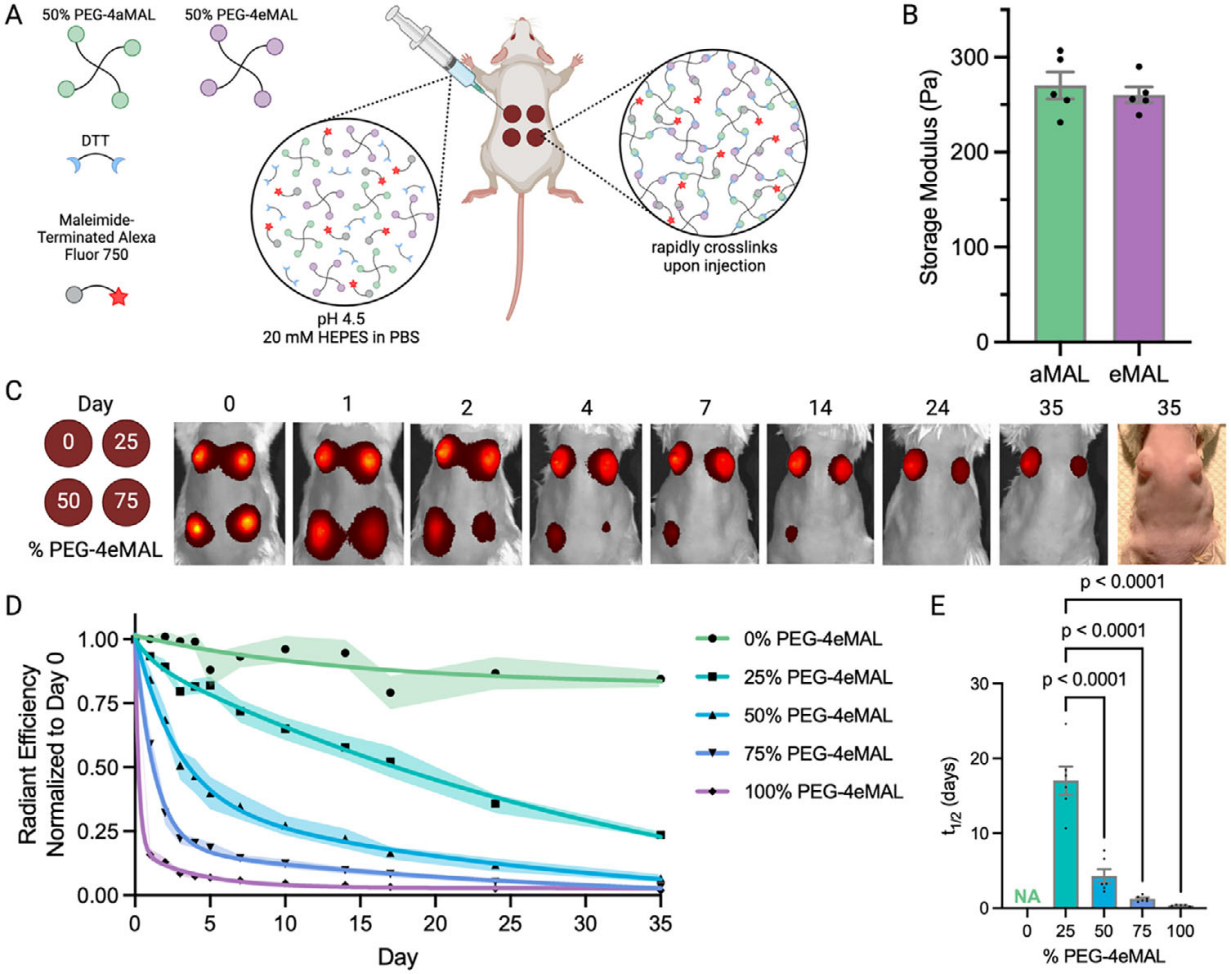
In vivo degradation kinetics of PEG‐4MAL hydrogels. A) Schematic of hydrogel fabrication with PEG‐4MAL (aMAL and eMAL) macromer, DTT crosslinker, and a near‐infrared fluorophore. The components were mixed prior to injection into the dorsal subcutaneous pocket of mice. B) A subset of hydrogels (*n* = 5/group) were harvested from the subcutaneous pocket 30 min after injection, swollen, and evaluated by rheology. Data presented as mean ± s.e.m. and analyzed with unpaired *t*‐test (*p* = 0.56). C) PEG‐4aMAL and PEG‐4eMAL were combined in various ratios (0%, 25%, 50%, 75%, and 100% eMAL) prior to subcutaneous injection (*n* = 5–6 gels/group) and fluorescence monitored for 35 days. Representative fluorescence images or photos of mouse dorsum following hydrogel injection. D) Radiant efficiency (normalized to day 0) of fluorescent hydrogels and exponential decay curve‐fit demonstrate tunability of degradation kinetics. Data presented as mean ± s.e.m. (shaded area). E) Half‐life of hydrogels calculated from the normalized radiant efficiency. Data presented as mean ± s.e.m. and analyzed with one‐way ANOVA followed by Tukey's multiple comparison analysis.

We posited that increasing the PEG‐4eMAL/PEG‐4aMAL ratio increases the in vivo degradation rate. 5 w/v% PEG (0%, 25%, 50%, 75%, and 100% PEG‐4eMAL) hydrogels crosslinked with DTT were synthesized for this experiment. A near‐infrared dye, maleimide‐terminated Alexa Fluor 750 (AF750), was incorporated into the network to allow for longitudinal fluorescence tracking of polymer degradation with an in vivo imaging system (IVIS). Male Balb/c mice received subcutaneous injections of hydrogels (5 w/v%) at four dorsal sites, each containing a different PEG‐4eMAL/PEG‐4aMAL formulation (**Figure** **4**A). Published studies indicate that these subcutaneous sites provide independent immune local microenvironments that allow for direct biomaterial comparisons in the same subject.^[^ ^16^ ^]^ All hydrogel formulations fully gelled in the subcutaneous space <1 min. A subset of hydrogels not including an adhesive ligand or AF750 dye was explanted 30 min post‐injection, swollen, and analyzed by rheometry (frequencies ranging from 1 to 10 Hz). The storage modulus between PEG‐4aMAL and PEG‐4eMAL hydrogels was equivalent, indicating no differences in in situ polymerization (Figure 4B). Representative images of fluorescence signal over 35 days demonstrate a faster decrease in fluorescent signal in correlation with higher PEG‐4eMAL content, and a visible difference in the hydrogel presence in the dorsal region (Figure 4C). Quantification of radiant efficiency of each site and the corresponding hydrogel half‐life exhibited PEG‐4eMAL content‐dependent differences in fluorescence signal, with faster loss of fluorescence signal with increasing PEG‐4eMAL content (Figure 4D,E). These results demonstrate increasing in vivo hydrogel degradation with increasing PEG‐4eMAL content.

We next explored the effect of PEG‐4MAL polymer density on in vivo degradation kinetics, utilizing a 50% PEG‐4eMAL macromer content formulation. Hydrogels were synthesized as described above while varying the PEG‐4MAL polymer density from 4% to 8% (w/v). Increasing polymer density slowed down the degradation rate, as indicated by the increased hydrogel half‐life (Figure S3, Supporting Information). We note that varying the % content of PEG‐4eMAL in the macromer backbone provided greater control over in vivo degradation rate compared to varying PEG‐4MAL polymer density.

### 2.5 PEG‐4eMAL/PEG‐4aMAL Ratio Modulates Local Immune Cell Responses

**Figure 5:**
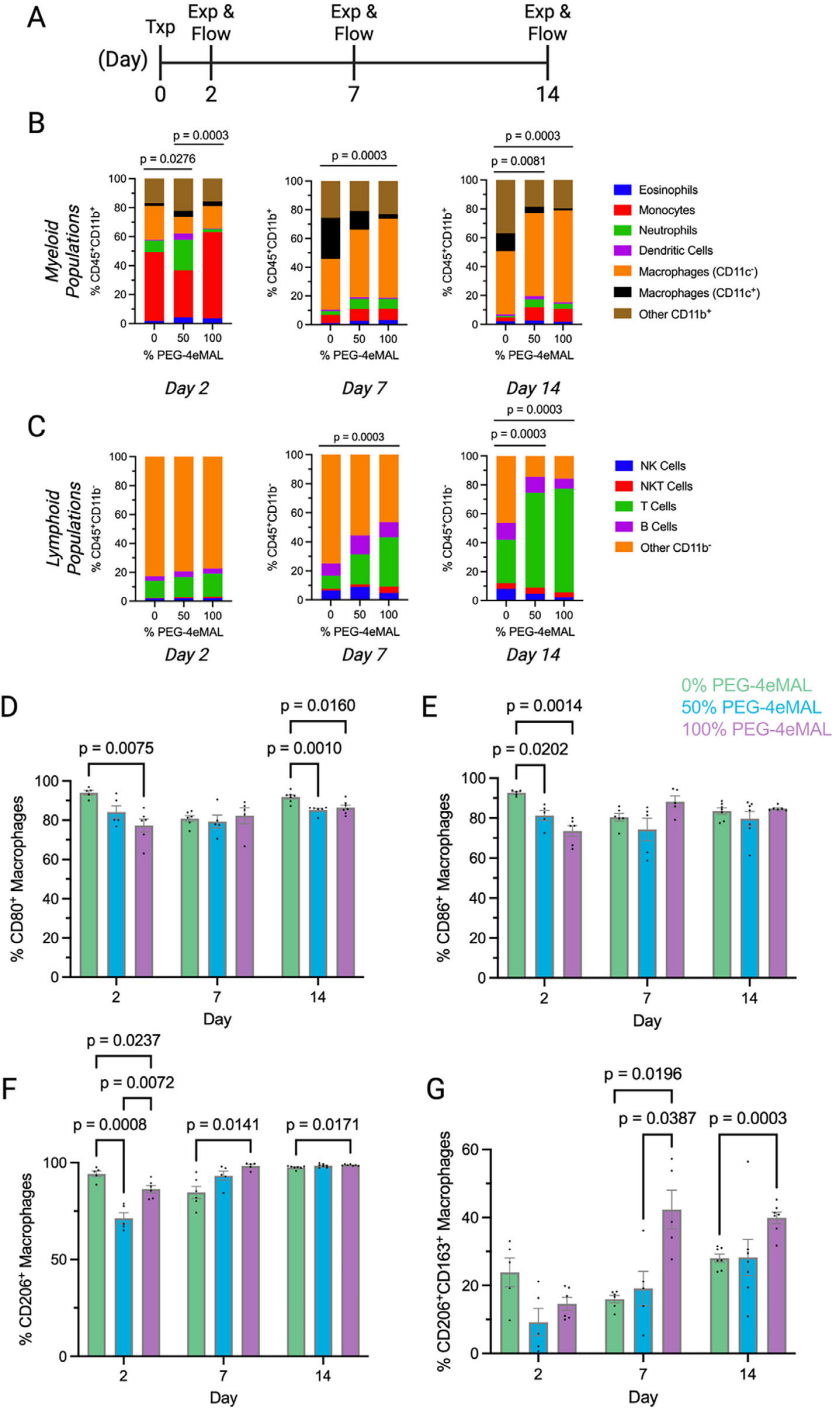
Immune cell profile after subcutaneous injection of PEG‐4MAL hydrogels. A) Timeline of subcutaneous hydrogel injection and takedown points for flow cytometry analysis. Distributions of B) myeloid and C) lymphoid immune cells in the subcutaneous explants across the three timepoints and different PEG‐4MAL formulations (*n* = 5/condition per timepoint). Data presented as fractional plots and analyzed with Chi‐square test followed by pairwise‐comparisons using Fisher's exact test, adjusted *p*‐values indicated on the plots. D) CD80^+^, E) CD86^+^, F) CD206^+^, and G) CD206^+^CD163^+^ macrophage populations calculated across PEG‐4MAL formulations over time. Data presented as mean ± s.e.m. with all individual points and analyzed with two‐way ANOVA.

We evaluated the effects of PEG‐4aMAL/PEG‐4eMAL ratio on local immune cell recruitment, as the hydrogel degradation rate could influence host responses to implanted biomaterials. Hydrogels synthesized using 0%, 50%, and 100% PEG‐4eMAL macromer, functionalized with 1.0 mm RGD and crosslinked with DTT, were injected subcutaneously in the same way as in the in vivo degradation study. At 2‐, 7‐, and 14‐days post‐transplantation, the injection sites and surrounding subcutaneous tissue were explanted and immune cells were isolated for analysis with quantitative spectral flow cytometry (**Figure** **5**A). Immune cells of interest and antibodies used are detailed in supplemental information (Table S1, Supporting Information).

Live single cells were identified and then split into either CD45^+^CD11b^+^ (myeloid) or CD45^+^CD11b^–^ (lymphoid) populations. Validated antibodies and appropriate Fluorescence‐Minus‐One (FMO) controls and gating strategies were used. Immune cell types were determined using the gating strategy denoted in supplemental information (Figure S4, Supporting Information).^[^ ^50^ ^]^ Myeloid cell populations showed a higher frequency of monocytes and neutrophils on day 2, and a higher presence of macrophages by day 7 and 14, consistent with the trajectory of innate immune responses.^[^ ^51^, ^52^ ^]^ Additionally, the presence of scaffold‐associated macrophages (F4/80^+^CD11c^+^, SAMs) was higher in 0% PEG‐4eMAL hydrogels, indicating that the lack of degradation increased SAMs to the local site (Figure 5B).^[^ ^53^ ^]^ Analysis of lymphoid cells indicated that gels with higher PEG‐4eMAL content induced higher T cell yet lower NK cell presence at the implant site (Figure 5C), the mechanisms of which will need to be examined in future studies. Additional data of myeloid and lymphoid cell distributions is presented in the supplemental information (Figure S5, Supporting Information).

We next investigated macrophage populations to assess whether the hydrogels were promoting inflammatory or regenerative environments. Once all F4/80^+^ macrophages were identified, populations of CD80^+^, CD86^+^, CD206^+^, and CD206^+^CD163^+^ were determined across hydrogel conditions and time points, and graphs were plotted as the subpopulation percentages of total macrophages. Results indicate that the nondegradable hydrogels (0% PEG‐4eMAL) induced recruitment of more CD80^+^ and CD86^+^ macrophages, and thus a more pro‐inflammatory microenvironment (Figure 5D,E). In contrast, hydrogels with 100% PEG‐4eMAL macromer attracted significantly higher pro‐regenerative CD206^+^ and CD206^+^CD163^+^ macrophages compared to its non‐degradable counterpart (Figure 5F,G). These results demonstrate a correlation between in vivo hydrogel degradation and macrophage polarization at the site of injection, potentially due to the sustenance of a pro‐inflammatory state, ultimately leading to chronic inflammation, when a biomaterial implant is non‐degradable.^[^ ^54^, ^55^ ^]^

## 3 Discussion

A critical property of synthetic hydrogels for biomedical applications is in vivo degradability, as this directly impacts host responses and tissue repair.^[^ ^18^, ^56^, ^57^, ^58^, ^59^ ^]^ Whereas considerable efforts have focused on protease‐degradable hydrogels that assimilate with the tissue environment,^[^ ^27^, ^60^, ^61^ ^]^ tunability of degradation is difficult to attain. In this study, we describe a PEG‐4MAL hydrogel system that combines hydrolytically‐degradable PEG‐4eMAL macromer with nondegradable PEG‐4aMAL macromer in stoichiometric ratios to tune in vivo degradability. We present a family of PEG‐4eMAL and PEG‐4aMAL hydrogels with equivalent mechanical properties but increased in vitro and in vivo degradation rate simply by increasing the PEG‐4eMAL content. PEG‐4eMAL/PEG‐4aMAL hydrogels supported high viability of embedded human cells. Notably, the ratio of PEG‐4eMAL/PEG‐4aMAL modulated local immune cell recruitment when implanted in the subcutaneous space. These results establish the use of PEG‐4eMAL/PEG‐4aMAL hydrogels as a hydrolytically degradable platform to tune in vivo degradation and immune responses.

Prior work on hydrolytically degradable synthetic hydrogels has predominantly centered around hydrolytically degradable crosslinkers.^[^ ^21^, ^22^, ^23^, ^24^, ^62^ ^]^ However, a study on hydrolytically degradable hyaluronic acid showed the potential of incorporating an ester group into the polymer backbone, offering another means of tuning degradation.^[^ ^20^ ^]^ Our group reported the effects of an ester linkage in 4‐arm PEG‐norbornene (PEG‐4NB) hydrogels on in vivo degradation, showing that the hydrogels degraded rapidly (within hours) compared to the long‐term stability of hydrogels with an amide linkage.^[^ ^35^ ^]^ Furthermore, we previously utilized a protease‐degradable peptide to crosslink PEG‐4aMAL and PEG‐4eMAL macromers into the hydrogel network.^[^ ^16^ ^]^ This hydrogel was used to deliver murine mesenchymal stromal cells to repair cutaneous wounds in diabetic mice. A limitation of this study was the application of hydrogels that were susceptible to both proteolytic and hydrolytic degradation, which confounded the degradation rate of the material. The present study focuses on engineering hydrogels with tunable in vivo degradation rates by varying the ratio of PEG‐4aMAL/PEG‐4eMAL macromers crosslinked with nondegradable DTT. The use of DTT crosslinker, in contrast to the protease‐degradable peptide, restricts the degradation mechanism to the ester‐containing macromer and results in a family of hydrogels with tunable in vivo degradation rates. The cell viability and spreading results in the present study are consistent with those of studies in hyaluronic acid hydrogels with ester groups. In both cases, human mesenchymal stromal cells spread more with a higher proportion of macromer with ester groups, supporting the conclusion that hydrolysis allows for faster degradation of the hydrogels and thus better cell spreading, which is supported in published literature across multiple regenerative medicine applications involving natural and synthetic biomaterials.^[^ ^6^, ^20^, ^43^, ^44^, ^45^, ^46^, ^63^, ^64^ ^]^

We hypothesized that PEG‐4eMAL content significantly hastens in vivo degradation and provides a facile mechanism to tune in vivo degradation rate. Indeed, as the PEG‐4eMAL content increases, the in vivo degradation rate increased. Additionally, for a fixed PEG‐4eMAL/PEG‐4aMAL ratio, increases in polymer density decreased in vivo degradation rate. Therefore, the PEG‐4eMAL/PEG‐4aMAL hydrogel system offers a simple method of finetuning degradation and optimizing PEG hydrogels to specific applications. Recent work from our group has shown that the PEG‐4eMAL hydrogels enabled murine MSC engraftment in subcutaneous wound healing,^[^ ^16^ ^]^ and it would be valuable to investigate other cells and transplant sites that could benefit from being delivered in a tunable, hydrolytically degradable gel.

We posited that changes in in vivo degradation rate associated with PEG‐4eMAL/PEG‐4aMAL ratio impact immune cell recruitment. We reported that hydrolytically degradable PEG‐4MAL microgels can be formulated by incorporating ethylene glycol bis(mercaptoacetate) (EGBMA) as an ester‐containing crosslinker. By changing the concentration of EGBMA to increase the number of ester‐containing crosslinks, microgel swelling, degradation, and leukocyte recruitment could be tuned.^[^ ^62^ ^]^ Whereas this prior work found that multiple myeloid and lymphoid cell populations decreased with the incorporation of EGBMA, the present study showed increases in T cell populations with the incorporation of the ester linkage. The differences in immune response could be due to differences in in vivo degradation due to differences in surface area/volume ratio^[^ ^65^ ^]^ or density of ester‐containing crosslinks between microgels and bulk hydrogels. A slower degradation rate is associated with increased T cell responses.^[^ ^66^, ^67^ ^]^ Taken together, these results establish the use of PEG‐4eMAL/PEG‐4aMAL hydrogels as a hydrolytically degradable platform to tune in vivo degradation and immune responses.

## 4 Experimental Section

### Hydrogel Synthesis

Amide‐linked PEG‐4aMAL (>95% degree of functionalization, 20 kDa, Laysan, USA) and custom‐synthesized ester‐linked PEG‐4eMAL (97% degree of functionalization/substitution, 96.9% purity, 19.6 kDa, Jenkem, China) were utilized for hydrogel synthesis. For in vitro and in vivo studies (unless otherwise noted), PEG‐4eMAL and PEG‐4aMAL (or the ratio mix of the two macromers) hydrogels were prepared at 5.0% (w/v) macromer concentration, 1.0 mm RGD peptide (GRGDSPC, AAAPTEC; custom peptide, USA), and 4.8 mm DTT (Invitrogen; 15 508 013, USA) or 4.25 mm VPM peptide (GCRDVPMSMRGGDRCG, Genscript; custom peptide, China); all are mixed in 20 mm HEPES (1m stock [Corning; 25‐060‐CI, USA] diluted in PBS with Ca^2+^/Mg^2+^ [Corning; 21‐030‐CM, USA]) in PBS (without Ca^2+^/Mg^2+^, Corning; 21‐040‐CM, USA) at pH 5.5–6.0. Crosslinker concentration was calculated by stoichiometrically balancing the number of unreacted maleimides on the PEG‐4MAL macromers with the number of free thiols (2 per crosslinker). Additional thiol‐ or maleimide‐containing components incorporated into the hydrogel (adhesive peptide, fluorophore) were factored into the overall thiol‐maleimide stoichiometric balance, and crosslinker concentration adjusted accordingly.

Hydrogel precursors (PEG macromer, RGD and DTT) were prepared at 2X the final concentration and mixed at 1:1 ratio to achieve the final concentration. For eMAL/aMAL hydrogels, a ratio of eMAL and aMAL were mixed prior to mixing with DTT. Macromers and crosslinkers were set on ice 15 min prior to casting the hydrogels to slow down the crosslinking reaction and allow for more mixing time. Mixing is performed by rapid pipetting for ≈5–7 s, ensuring care not to introduce bubbles. Hydrogels were cast on Parafilm for 20–30 min at room temperature before swelling in PBS without Ca^2+^/Mg^2+^ (Corning; 21‐040‐CM, USA) at 25 °C.

### Hydrogel Rheology

PEG‐4eMAL and PEG‐4aMAL hydrogels (8 µL) were mixed with DTT, cast on Parafilm in a dome shape, and subsequently swollen in PBS (without Ca^2+^/Mg^2+^) overnight at 25 °C. Hydrogel macromer density ranged from 3% to 10% (w/v) for PEG‐4aMAL and PEG‐4eMAL hydrogels, and macromer ratio was adjusted from 0:1 to 1:0 for the 5% (w/v) condition. Rheological measurements were obtained using a 10 mm diameter, 2° cone on an MCR 302 rheometer (Anton Paar). Hydrogel samples were individually loaded onto the plate, the cone was lowered, excess hydrogel was trimmed using a blade, and the hydrogel was hydrated with PBS (without Ca^2+^/Mg^2+^) for the duration of the test. The storage modulus (*G*’) was recorded as the average *G*’ modulus over an oscillatory frequency range (1–10 Hz) at 1.5% strain and 25 °C (the strain was determined to be within the linear viscoelastic range by using an oscillatory strain amplitude sweep). For explanted samples, hydrogels were explanted from the subcutaneous space of the mouse dorsum 30 min after injection. The hydrogels were washed in dH_2_O 3X (30 min each), swollen overnight in dH_2_O, and rheological properties were recorded as described above.

### Hydrogel FTIR

PEG‐4eMAL and PEG‐4aMAL hydrogels (25 µL; 5% w/v) were mixed with DTT, cast on Parafilm, and swollen in dH_2_O overnight at 25 °C. Hydrogels were washed 2X with dH_2_O for 30 min each on an orbital shaker (100 rpm) and lyophilized. Following lyophilization for 72 h, FTIR transmission spectra were acquired with a Shimadzu IRPrestige 21 instrument. The amide and ester peaks were identified using the Shimadzu software.

### In Vitro Degradation

5% (w/v) 20 kDa PEG‐4MAL (PEG‐4aMAL or PEG‐4eMAL), 1.0 mm RGD, and DTT were mixed to form 80 µL gels in 24‐well nontissue culture treated plates (*n* = 90/gel condition) (Corning; 351147, USA). After gel polymerization, 1 mL buffer was added to the wells to begin swelling; media was either PBS (without Ca^2+^/Mg^2+^) (*n* = 30), 16% FBS (Gibco; 12 662 029, USA) in PBS (*n* = 30), or 100% FBS (*n* = 30). Day 0 reference gels were swollen in media at 37 °C for 1 h, rinsed 3X in dH_2_O for 1 h to remove residual serum components and salts, frozen at −80 °C for at least 30 min, and lyophilized for 24 h. Gels at their respective timepoints were washed, frozen, and lyophilized to track degradation over time. Dry masses were measured 24 h after lyophilization.

For room temperature (25 °C) experiments, hydrogels (*n* = 25/condition) were transferred to a 12‐well plate (*n* = 1 hydrogel/well) (Corning; 351143, USA), immersed in 3 mL PBS (Gibco; 14 190 144, USA), sealed with Parafilm to prevent evaporation, and stored at 25 °C for up to 28 days. At various timepoints (day 1, 4, 7, 14, 28), hydrogels (*n* = 5/condition) were removed, rinsed 3X in dH_2_O for 1 h to remove residual serum components and salts, frozen at −80 °C, and lyophilized for 48 h. The mass in milligrams of the lyophilized hydrogels was recorded as the dry mass.

For serum degradation experiments, hydrogels were prepared as above and stored in PBS (without Ca^2+^/Mg^2+^) at room temperature for 24 h to swell prior to incubation. Blood was collected from Balb/c mice (≈1–1.5 mL/mouse) via cardiac puncture into SST tubes (BD; 365 967, Puerto Rico), clotted for 45 min at 25 °C, and spun at 1200g for 15 min. Serum was collected and filtered using 0.45 µm centrifuge filters (Corning; 8162, USA). Half the serum was designated as fresh, and the other half was heat inactivated by immersing the tube containing serum in a 56 °C stirred water bath for 30 min. Heat inactivated FBS (HI‐FBS) was commercially sourced (Corning; 35‐011‐CV, Costa Rica/USA). Hydrogels (*n* = 2/condition) were transferred to a 24‐well plate (*n* = 1 hydrogel/well) and immersed in serum conditions (1.5 mL/well) for 24 h in incubator at 37 °C. Following incubation, hydrogels were rinsed in PBS, excess moisture removed, imaged (iPhone), and the mass was recorded as the wet mass.

### In Vitro Cell Viability

Human mesenchymal stromal cells (provided as frozen aliquots of de‐identified samples obtained under IRB‐approved protocols by the NIH Resource Center at Texas A&M University, College Station, TX) were thawed and expanded using a complete culture media (CCM) comprised of MEM‐Alpha media with no nucleosides (Gibco; 12 561 056, USA), MSC‐qualified FBS (Gibco; 12 662 029, USA), L‐Glutamine 200 mM (Gibco; 25 030 081, USA/UK), and Pen/Strep 10 000 U mL^−1^ (Gibco; 15 140 122, USA). PEG‐4MAL hydrogels (20 µL) were fabricated to achieve final concentrations of 5% (w/v), 1.0 mm RGD, 1×10^6^ hMSCs mL^−1^, and were crosslinked either with VPM or DTT. All gels were formed in nontissue culture treated 24‐well plates and fed with 1 mL CCM/well. At the imaging timepoints, gels were moved to a different 24‐well plate to ensure imaged cells were within the gel. One gel per condition was incubated in 70% isopropyl alcohol for 15 min to form a dead cell control. Calcein AM (Invitrogen; C1430, USA) and Ethidium Homodimer‐1 (Invitrogen; E1169, USA) were mixed in CCM and added to each gel. Gels were incubated at 37 °C and 5% CO_2_ for 15 min on a shaker (70 rpm). Gels were imaged on a Zeiss 700A Laser Scanning Confocal Microscope; images were processed on Zen Black and Zen Blue and later analyzed on FIJI.

### In Vivo Hydrogel Degradation

All animal experiments were performed with the approval of the Georgia Tech Animal Care and Use Committee with veterinary supervision (protocol A100318) and within the guidelines of the Guide for the Care and Use of Laboratory Animals. Male BALB/cJ mice (12–15 weeks old) were purchased from Jackson Laboratories (#000651, USA). PEG‐4eMAL and PEG‐4aMAL hydrogel components were prepared individually at higher initial concentrations before mixing to achieve a final concentration of 5% (w/v) PEG macromer, 4.8 mm DTT (see “Hydrogel synthesis” section for discussion of crosslinker concentration), and 35 µm Alexa Fluor 750‐maleimide (AF750‐MAL) (Invitrogen; A30459, USA). The volumetric mixing ratio for these components was 4:1:5 (PEG:AF750‐MAL:DTT), so the initial concentrations of each component were 2.5X (12.5% w/v), 10X (350 µm), and 2X (9.6 mm), respectively. PEG and DTT were reconstituted in 20 mm HEPES (Gibco; 15 630 080, USA/UK) in PBS (without Ca^2+^/Mg^2+^) adjusted to pH 4.3 with 1 m HCl. AF750‐MAL was reconstituted in DMSO at 1 mg mL^−1^ and diluted to an initial concentration 0.35 mm (0.48 mg mL^−1^) with PBS (without Ca^2+^/Mg^2+^). All components were maintained on ice prior to hydrogel fabrication. In some iterations, DTT was replaced with the cysteine‐terminated protease‐degradable crosslinker VPM. In some experiments, the PEG hydrogel density was adjusted between 4% and 8% (w/v), and the crosslinker concentration was adjusted according to stoichiometric balance of thiol‐maleimides.

For hydrogel implantation, mice were anesthetized with isoflurane, fur from the dorsal region was shaved and further removed with a chemical depilatory (Nair), and the site was cleaned with 70% isopropanol and chlorhexidine. The mouse dorsum was visually subdivided into four quadrants for injections of hydrogels. In an Eppendorf tube on ice, the PEG and AF750‐MAL were first mixed (20 µL + 5 µL), followed by rapid mixing with DTT (25 µL) using a quick vortex (≈2 s). The fully mixed hydrogel (50 µL) was withdrawn into an insulin syringe (BD; 328 279, USA) and injected into the subcutaneous space of the mouse dorsum. The hydrogel rapidly crosslinks upon injection due to interstitial fluids rapidly buffering the thiol‐maleimide reaction upon injection. The fluorescent intensity of the hydrogel was measured using the IVIS Spectrum CT (Perkin Elmer). The radiant efficiency [p/s/sr]/[µW cm^−2^] within the region of interest (ROI) was recorded at selected time points between day 0 and 35.

### Analysis of Immune Cell Recruitment

Male 11–12‐week‐old BALB/c mice (Jackson Laboratories) were used to assess immune responses to PEG‐4eMAL hydrogels. To prepare for subcutaneous injection, the dorsal subcutaneous region was shaved but not applied with Nair, as it can cause irritation and skin thickening, thus interfering with results. Hydrogels were prepared and injected the same way as the in vivo degradation experiment, and nylon sutures were tied above and below the injection sites to mark their locations to better identify when explanted.

At the takedown (2‐, 7‐, and 14‐days post‐transplant), injection sites and the surrounding subcutaneous tissue were explanted using a 12 mm biopsy punch. Tissue samples were cut into fine fragments and digested in a buffer containing Rosewell Park Memorial Institute (RPMI) 1640 media (Gibco; 11 875 093, USA), 2.5 U mL^−1^ Dispase II (ThermoFisher; 17 105 041, USA) and 0.2% Type II Collagenase (Worthington Biochemical Corp.; LS004176, USA) at 37 °C for 1 h. The homogenized tissue was filtered through a 35 µm cell strainer (Falcon; 352 235, USA) and washed with PBS to remove debris. Red blood cell (RBC) lysis buffer (Biolegend; 420 301, USA) was used to lyse red blood cells from the mixture, and the remaining cell pellet was washed with fluorescence‐activated cell sorting (FACS) buffer— Hank's Balanced Salt Solution (HBSS) (Gibco; 14 175 095, USA), 0.5% bovine serum albumin (BSA) (Millipore Sigma; A9418), and 2 mm ethylenediaminetetraacetic acid (EDTA) (0.5 m stock, Invitrogen; AM9260G, USA)—and then PBS. Cells were first stained with the Fixable Blue Dead Cell Dye (ThermoFisher; L23105, USA) for 15 min, washed with FACS buffer, blocked with TruStain FcX PLUS (Biolegend; 156 604, USA), stained with all the remaining antibodies (Table S1, Supporting Information, all based in the USA), and finally fixed with Fixation Buffer (Biolegend; 420 801, USA). Data was collected with the Cytek Aurora spectral flow cytometer and analyzed with FlowJo.

### Statistical Analysis

All statistical analyses were performed on the GraphPad Prism 9 software. All bar plots are displayed as mean ± standard error of the mean (s.e.m.). Plots that are functions of time are depicted as mean (points) and s.e.m. (shaded region). Figure legends indicate the sample size and statistical tests in each experiment.

## Conflict of Interest

A.J.G., M.D.H., and K.E.M. are co‐inventors on a patent application filed by the Georgia Tech Research Corp. on the on‐chip 3D potency assay. The remaining authors declare no competing interests.

## Author Contributions

M.D.H. and S.K. contributed equally to this work. M.D.H, S.K., K.E.M, and A.J.G. conceived and designed the project. M.D.H. and S.K. designed and led the research, analysis, and experimental interpretation. M.D.H., S.K., K.E.M., A.L.T., S.W.L., G.B., and R.S.S. conducted experiments. M.D.H., S.K., and A.J.G. wrote the manuscript. A.J.G. supervised and funded the study.

## Supporting information



Supporting Information

## Data Availability

The data that support the findings of this study are available from the corresponding author upon reasonable request.
