# Supplementary material for: Synthetic Hydrogels Incorporating Hydrolytic/Nonhydrolytic Macromer Ratios Exhibit Improved Tunability of In Vivo Degradation and Immune Responses
Source: Adv Healthc Mater. 2025 Oct 1;15(3):e02475. doi: 10.1002/adhm.202502475 (PMC12817107; doi:10.1002/adhm.202502475)
Supplement: Supplementary file 1 — Supporting Information [file ADHM-15-0-s001.pdf]

ADVANCED HEALTHCARE MATERIALS

Supporting Information

for *Adv. Healthcare Mater.*, DOI 10.1002/adhm.202502475

Synthetic Hydrogels Incorporating Hydrolytic/Nonhydrolytic Macromer Ratios Exhibit Improved Tunability of In Vivo Degradation and Immune Responses

*Michael D. Hunckler, Sophia Kioulaphides, Karen E. Martin, Angelica L. Torres, Graham F. Barber, Stephen W. Linderman, Rebecca S. Schneider and Andrés J. García**

Supporting Information

Synthetic Hydrogels Incorporating Hydrolytic/Non-Hydrolytic Macromer Ratios Exhibit Improved Tunability of In vivo Degradation and Immune Responses

*Michael D. Hunckler, Sophia Kioulaphides, Karen E. Martin, Angelica L. Torres, Graham F. Barber, Stephen W. Linderman, Rebecca S. Schneider, Andrés J García**

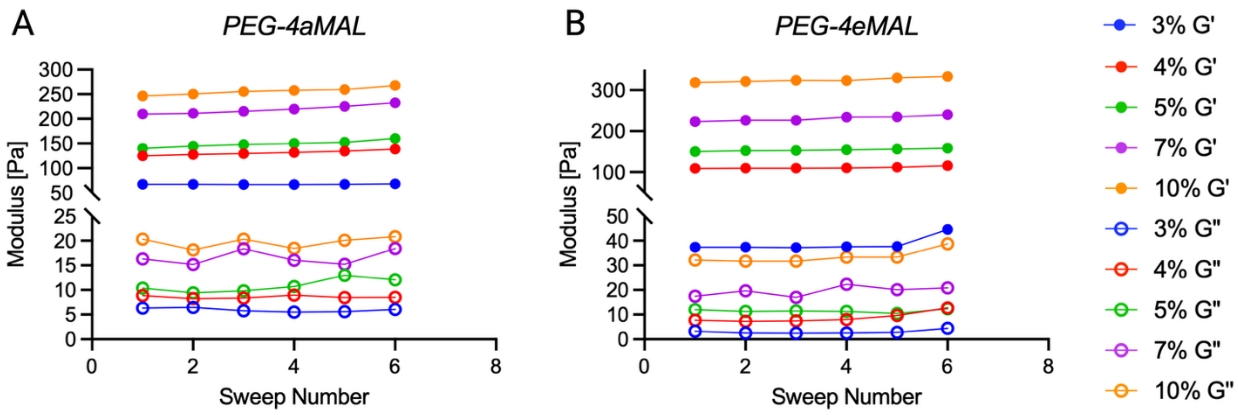

Figure S1. Frequency sweeps of PEG-4aMAL and PEG-4eMAL hydrogels in rheology. Full frequency sweeps of PEG-4MAL hydrogels ranging from 3 to 10% (w/v), specifically of A) 100% PEG-4aMAL and B) 100% PEG-4eMAL hydrogels.

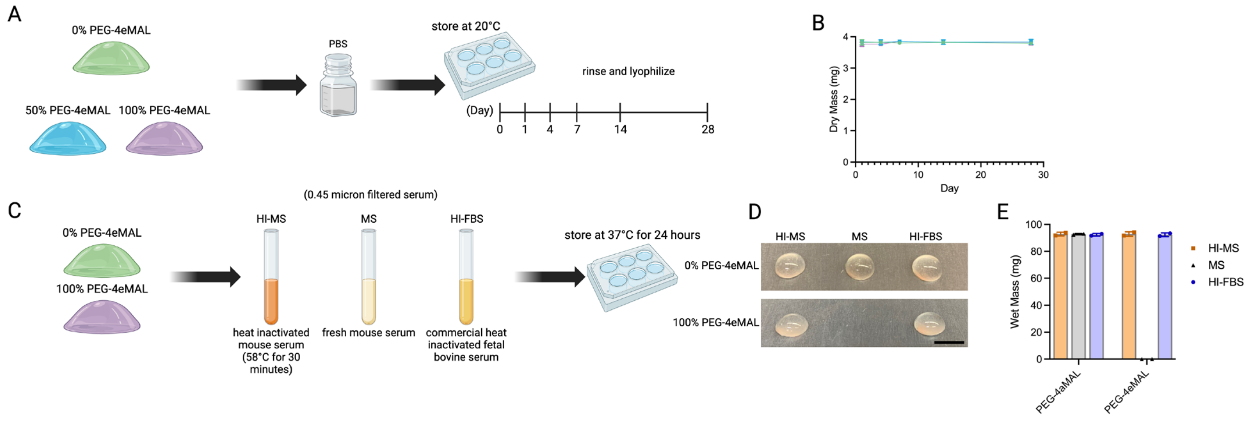

Figure S2. *In vitro* degradation of PEG-4MAL hydrogels in different serum-containing media. A) Schematic of hydrogel degradation experiment in PBS. 5% PEG-4MAL hydrogels (75 µL) with 100% PEG-4aMAL, 50/50% PEG-4aMAL/PEG-4eMAL, or 100% PEG-4eMAL were crosslinked with DTT and swollen in PBS (without Ca^{2+}/Mg^{2+}, pH 7.4) at 25°C. B) At various timepoints, the hydrogels were removed from PBS, rinsed (dH_2O for 6 hours), and lyophilized. Hydrogels did not exhibit degradation up to day 28 (n = 5 gels per group per timepoint; two-way ANOVA, p = 0.3). C) Schematic of hydrogel degradation experiment in serum: heat inactivated fresh mouse serum (HI-MS), fresh mouse serum (MS), and commercial heat inactivated fetal bovine serum (HI-FBS). 5% PEG-4MAL hydrogels (100 µL) were crosslinked with DTT and swollen in PBS prior to incubation in serum at 37°C for 24 hours. D-E) After 24 hours, PEG-4eMAL hydrogels completely degraded in fresh non-heat inactivated mouse serum (MS), while all other conditions were equivalent (scale bar = 1 cm).

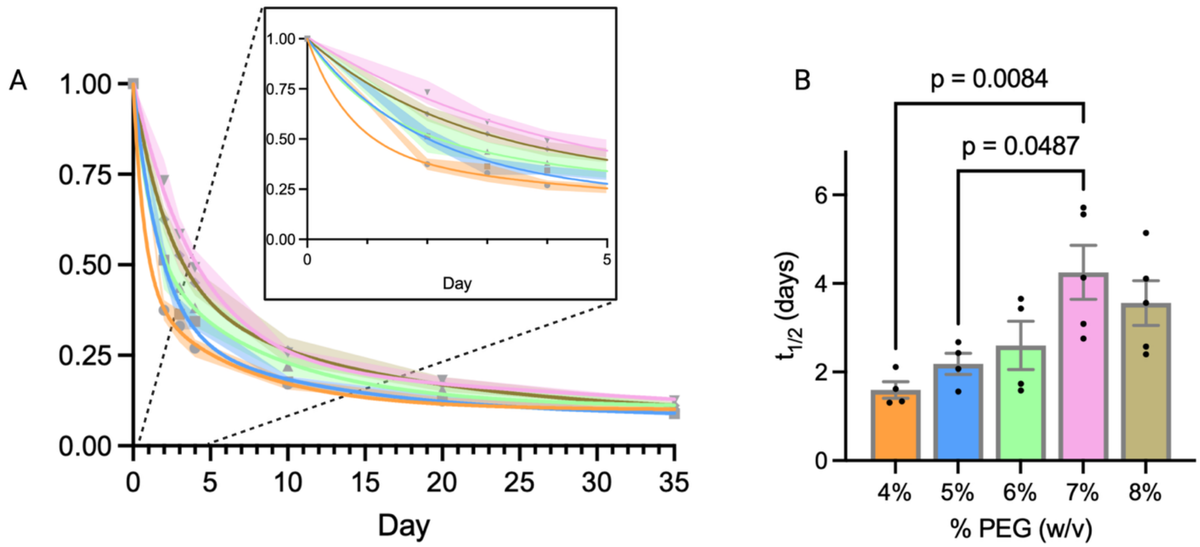

Figure S3. *In vivo* degradation kinetics of PEG-4MAL (50% PEG-4aMAL + 50% PEG-4eMAL) hydrogels with a range of PEG polymer density. A) PEG-4MAL (50% PEG-4aMAL + 50% PEG-4eMAL) hydrogels were fabricated at a range of PEG polymer density (4%, 5%, 6%, 7%, 8% w/v) immediately prior to subcutaneous injection (n = 5-6 gels/group) and fluorescence monitored for 35 days. Normalized radiant efficiency of fluorescent hydrogels and exponential decay curve fit demonstrate some tunability of degradation kinetics with initial polymer density. Inset included of days 0-5 of imaging. Data presented as mean (points) ± s.e.m. (shaded area). B) Half-life of hydrogels calculated from the normalized radiant efficiency. Data presented as mean ± s.e.m. and analyzed with one-way ANOVA followed by Tukey's multiple comparison analysis.

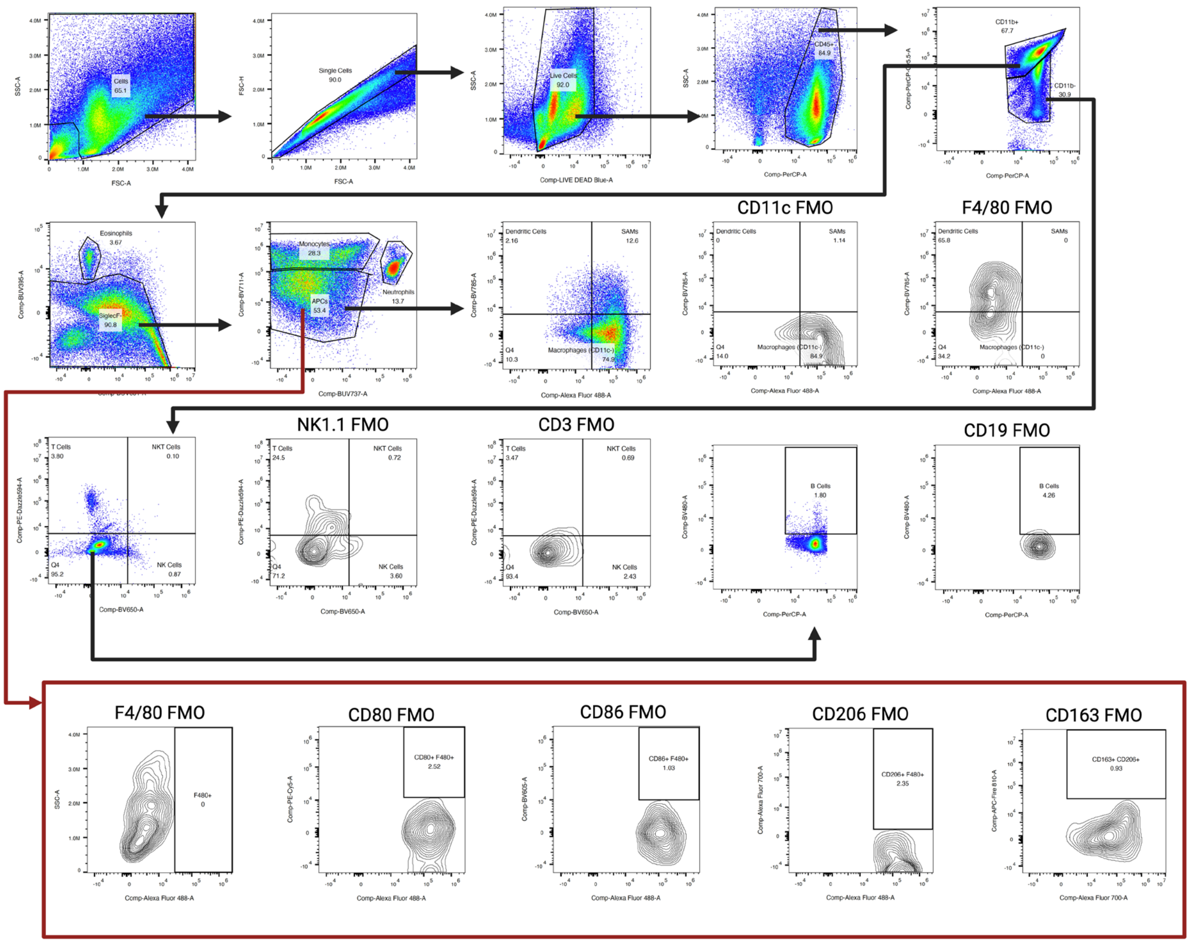

Figure S4. Gating strategy for flow cytometry analysis.

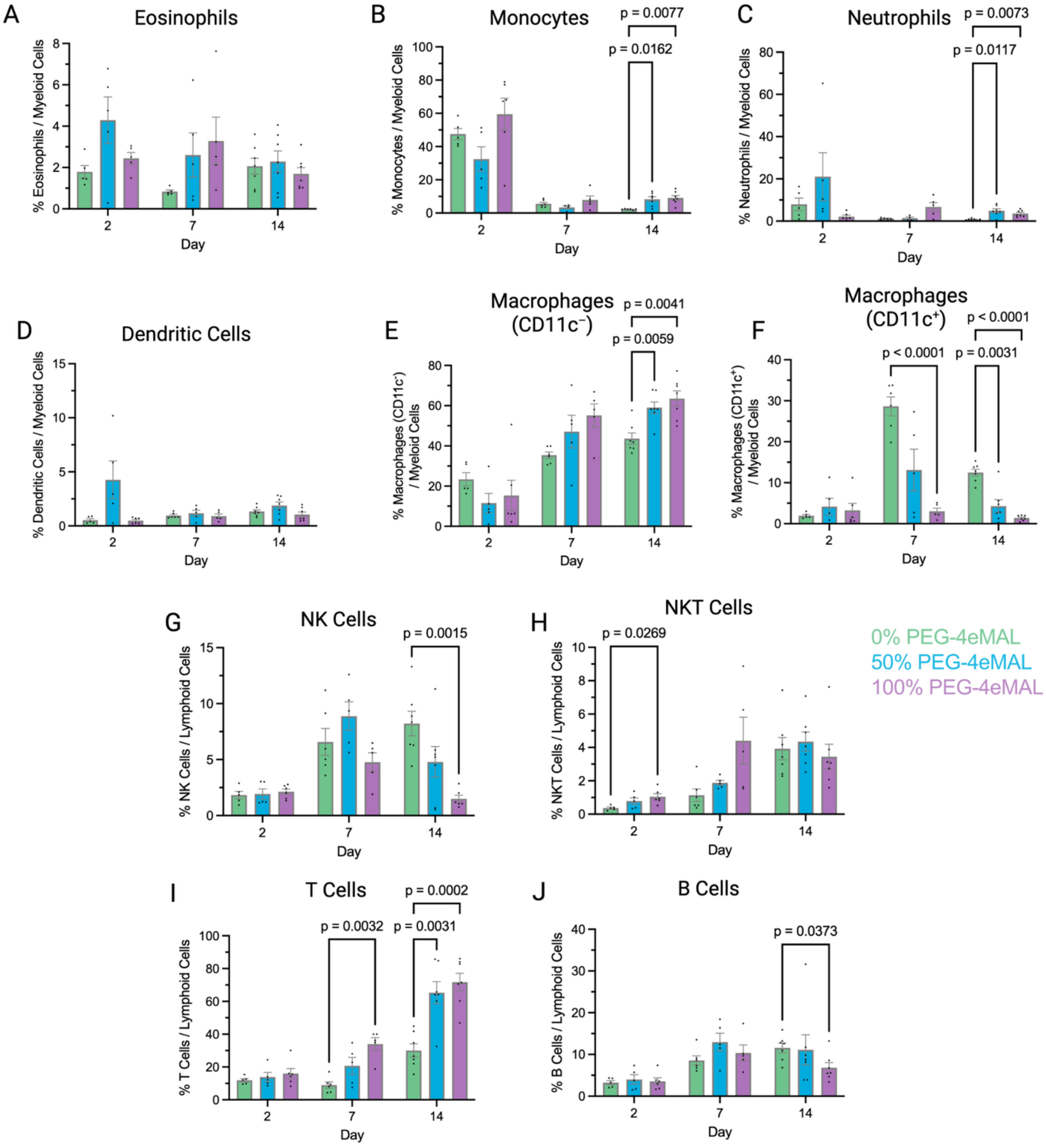

Figure S5. Myeloid and Lymphoid Cell Populations Over Time. Percent of A) eosinophils, B) monocytes, C) neutrophils, D) dendritic cells, E) $CD11c^-$, and F) $CD11c^+$ macrophages in the myeloid ($CD45^+CD11b^+$) population. Percent of G) NK cells, H) NKT cells, I) T cells, and J) B cells in the lymphoid ($CD45^+CD11b^-$) population. Data presented as bar plots with individual points (mean ± s.e.m.), outliers were identified and excluded, and plots were analyzed with Two-Way ANOVA.

Table S1. Antibodies for flow cytometry

Cell Type	Marker	Fluorochrome	Company	Catalog Number
Dead Cells	Live/Dead	Fixable Blue Dead Cell Dye	Thermo Fisher	L23105
Eosinophils	Siglec-F	BUV395	BD Biosciences	740280
MHC-II	MHC-II	BUV661	BD Biosciences	750280
Neutrophils	Ly6G	BUV737	BD Biosciences	741813
B Cells	CD19	BV480	BD Biosciences	566107
Inflammatory Macrophages	CD86	BV605	Biolegend	105125
NK Cells	NK1.1	BV650	Biolegend	108736
Monocytes	Ly6C	BV711	Biolegend	128037
Dendritic Cells	CD11c	BV785	Biolegend	117336
Macrophages	F4/80	Alexa Fluor 488	Biolegend	123120
Leukocytes	CD45	PerCP	Biolegend	103130
Myeloid Cells	CD11b	PerCP-Cy5.5	Biolegend	101228
T Cells	CD3	PE Dazzle 594	Biolegend	100246
Inflammatory Macrophages	CD80	PE-Cy5	Biolegend	104712
Regenerative Macrophages	CD206	Alexa Fluor 700	Biolegend	141734
Regenerative Macrophages	CD163	APC Fire 810	Biolegend	155321
